# Evaluation of an Oral Hygiene Education Program for Staff Providing Long-Term Care Services: A Mixed Methods Study

**DOI:** 10.3390/ijerph17124429

**Published:** 2020-06-19

**Authors:** Shang-Jung Wu, Chun-Chieh Wang, Shu-Chen Kuo, Shwn-Huey Shieh, Yueh-Juen Hwu

**Affiliations:** 1 Department of Nursing, Taichung Veterans General Hospital Puli Branch, Nantou 54552, Taiwan; ernr1191@gmail.com; 2 Department of Public Health, China Medical University, Taichung 40447, Taiwan; 3 Department of Internal Medicine, Taichung Veterans General Hospital Puli Branch, Nantou 54552, Taiwan; proteinmad@yahoo.com.tw; 4 Department of Eldercare, Central Taiwan University of Science and Technology, Taichung 40601, Taiwan; kuokuo460603@gmail.com; 5 Department of Nursing, Da Chien Health Medical System, Miaoli 36052, Taiwan; 6 Department of Health Services Administration, China Medical University, Taichung 40447, Taiwan; shshieh@mail.cmu.edu.tw; 7 College of Nursing in the Central Taiwan University of Science and Technology, Taichung 40601, Taiwan

**Keywords:** oral health, oral hygiene, long-term care, concurrent triangulation

## Abstract

Background: Oral hygiene is often neglected in clients receiving long-term care, suggesting that long-term care workers require formal oral hygiene education. Thus, the aim of this study was to investigate the effects of oral hygiene education on long-term care workers. Methods: This study utilized a mixed methods design. Eighty long-term care workers were recruited for participation in the oral hygiene education program, which employed three teaching methods: narration with multimedia presentation, demonstration, and teach-back. The effect of the education program on the participants’ level of oral hygiene knowledge, attitudes, and skills was measured using a structured questionnaire that was administered both pre- and post-delivery of the education program. Three months later, all participants submitted a self-report of their oral hygiene skills, and six participants completed a telephone interview. Quantitative data were analyzed using paired *t*-tests, and qualitative data were manually analyzed and coded. Results: Scores of oral hygiene knowledge (*p* < 0.001), attitudes (*p* = 0.001), and oral cleaning daily frequency for clients (*p* < 0.001), were significantly higher three months after undertaking the educational program. Conclusions: This preliminary study suggests that oral hygiene education may be effective in improving oral hygiene knowledge, attitudes, and skills among long-term care staff.

## 1. Introduction

The oral cavity is a potential reservoir for pathogens. Thus, poor oral hygiene facilitates microorganism-induced systemic diseases, such as pulmonary disease [1], cardiovascular disease [2], and dental plaque; the latter of which is significantly correlated with periodontal and systemic disease in humans [3]. Poor oral health can lead to the gradual impairment of swallowing ability, aggravation of cognitive impairment, and eventually results in malnutrition and social isolation [4,5,6]. Oral health has a significant impact on the ability of older adults to chew and swallow, and is of particular importance in individuals with dysphagia, who have an increased risk of aspiration of food remaining in the oral cavity after eating [7,8]. The prevalence of dysphagia in older adults residing in institutional long-term care facilities is as high as 68% [9]. Previous studies have shown that the provision of enhanced oral hygiene to frail clients in long-term care facilities can offer several benefits, including prevention of pneumonia and malnutrition, as well as improvement of systemic conditions [10,11]. Therefore, the oral hygiene of clients receiving long-term care services warrants additional attention.

Oral hygiene comprises part of the daily care of long-term care clients. Registered nurses, social educators, nursing assistants, and care workers are responsible for providing oral hygiene in long-term care facilities [12]. Although many oral hygiene protocols have been established in long-term care service institutions, they are neither sufficiently frequent nor thorough [13,14], and the oral hygiene of the clients remains suboptimal [13,15]. Low priority, lack of oral hygiene knowledge and skills, and insufficient oral health care training and education are factors that hinder care workers from performing daily oral care [16]. In addition, previous research exploring the feeding experiences of nursing assistants has shown that long-term care workers require additional knowledge and skills to provide adequate oral hygiene to clients with dysphagia [17].

Educational programs have been shown to not only increase knowledge, but also to improve the attitudes of long-term care workers toward oral hygiene [18]. Studies conducted by Garry [19] and Weening-Verbree et al. [20] have shown that increasing the level of knowledge and skills relating to oral hygiene among care workers is an effective strategy for improving the oral health of clients. Hence, receiving educational training may support long-term care workers in the delivery of optimal oral health care services.

Knowledge, Attitude and Practice (KAP) are three evidence-based structures used to understand professionals’ behavior and behavioral changes in oral care [21,22]. KAP regarding oral hygiene refers to knowledge as the “understanding of the topic regarding oral hygiene”, attitude as “feelings towards oral hygiene, as well as predetermined ideas towards oral hygiene”, and practice as “ways to demonstrate knowledge and attitudes through behavior towards oral hygiene [23,24]. Albrecht et al. [25] conducted a systematic review to assess the effects of oral hygiene educational interventions for nursing staff or residents on the maintenance or improvement of the oral health of nursing home residents, which involved educational sessions on oral hygiene for nursing staff (five trials) or for both staff and residents (four trials). The results showed that there was insufficient evidence to draw robust conclusion about the effects of oral hygiene educational interventions for nursing home staff and residents, and important measures such as staff knowledge and attitudes were rarely assessed.

The purpose of this study was to implement an oral hygiene education program, and to use a mixed methods design to investigate its effects on the knowledge, attitudes, and skills relating to oral hygiene among long-term care service staff. It was hypothesized that participants who received the oral hygiene education program would exhibit improved knowledge, attitudes, and skills relating to oral hygiene. The primary outcomes of this study were to assess the effectiveness of an oral hygiene education program on oral hygiene knowledge and attitudes among long-term care workers. The second outcome was to compare the behavioral changes in long-term care workers performing oral hygiene after completing the training program.

## 2. Materials and Methods

### 2.1. Study Design and Participants

The current study utilized a concurrent triangulation mixed-methods design that collected both qualitative and quantitative data. A pre/post-program assessment with one day of oral hygiene education, as well as a three-month pilot extension, was undertaken. The participants reported their performance of a three-month practical application, which included submitting various situational photos taken during practical application as well as filling in a questionnaire about daily oral cleaning frequency, products, and sites in order for the researchers to evaluate their behavior changes and then participants were presented with a certificate of completion. In addition, one-tenth of the participants were randomly selected using simple random sampling technique by a statistical expert, who was not a member of the research team, for a telephone interview to investigate their perceptions toward the oral hygiene education program in order to understand the changes in their beliefs, attitudes, and behaviors. The telephone interview was conducted by one researcher. A researcher-developed interview guide was used for the telephone interview, including: “Can you please tell me how you perceive the oral hygiene education program?”, and “Can you please tell me how the oral hygiene education program has affected your daily care?”

The study population comprised of employees working in long-term care service institutions (LTCSIs) in the Taichung municipality in Taiwan. Convenience sampling was used to recruit volunteers from an online environment to participate in a one-day oral hygiene education program. According to the long-term care service law in Taiwan, nurses must supervise nursing assistants [26]. In addition, because oral health is related to the holistic health of clients, long-term care workers, including social workers and administrators, should also equip themselves with knowledge and skills relating to oral hygiene care. Administrators play a pivotal role in coordinating and supporting the oral care services provided in long-term care facilities [27]. Therefore, this study also recruited administrators to participate in the oral hygiene education program. In total, 80 long-term care workers (such as nurses, nursing assistants, social workers, and administrators) participated in the program.

### 2.2. Oral Hygiene Education Program

The oral hygiene education program aimed to deliver oral hygiene knowledge to assist participants’ understanding of the relationship between oral hygiene and physical health, and to change their attitudes and behaviors toward oral hygiene. Eventually, the program aimed to help participants develop good oral hygiene skills for clients with, or without, dysphagia.

A one-day (i.e., eight-hour long) education program was developed in accordance with the oral healthcare teaching methods established by the Taiwanese Ministry of Health and Welfare [28]. The first four hours were dedicated to narration with multimedia presentations, which educated participants on the relationship between oral health and general health in older adults, as well as the prevention and treatment of oral disease (e.g., dry mouth, halitosis, and periodontal disease). The fifth hour was dedicated to oral care narration using dental models and oral hygiene care products, such as the Bass tooth brushing technique [29]; oral cleaning using dental floss; oral swabs and mouthwash; denture cleaning; gum cleaning; and tongue cleaning. The sixth hour involved demonstrations of oral hygiene skills, and the final two hours involved teach-back of oral hygiene skills. Each participant was required to pass a skills test of tooth brushing and flossing ability. The oral hygiene education program was delivered by four teachers, who were qualified as registered nurses with a master’s degree and over 10 years’ work experience in long-term care.

### 2.3. Questionnaire Design for Pre/Post-Program Assessment

A structured questionnaire (Table 1) was developed to assess the oral hygiene knowledge, attitudes, and skills of long-term care workers [30,31]. The questionnaire content and scoring method for the three parts are described below.

#### 2.3.1. Oral Hygiene Knowledge

The prevalence of periodontal disease among adults over 18 years old in Taiwan exceeds 90% [32], and severe periodontal disease in this demographic will lead to tooth loss. In addition, oral diseases can cause systemic health problems. Oral hygiene plays an essential role in exiting this harmful trajectory; therefore, the oral hygiene knowledge section of the questionnaire was designed with a focus on these issues [13,30]. This section consisted of 10 true–false items, which specifically covered the long-term care workers’ understanding of the health problems caused by poor oral hygiene, such as periodontal disease, denture care, and the function of toothbrushes and dental floss. One point was awarded for each correct answer, and wrong answers were awarded zero points. The scores ranged from 0 to 10 points.

#### 2.3.2. Oral Hygiene Attitudes

The section on oral hygiene attitudes consisted of the following three statements: “I will develop an individualized oral hygiene plan for clients”; “I know how to perform oral hygiene procedures for my clients every day”; and “I will try to improve the oral health status of my clients”. One point was awarded for agreement with each statement, and zero points were awarded for disagreement. Scores ranged from zero to three points, with higher scores indicating a more positive attitude toward oral hygiene. The use of a Likert-type scale may have produced vague data due to variation in the interpretation of responses [33].

#### 2.3.3. Oral Hygiene Skills

The oral hygiene skills section comprised a paper and pencil exercise in which participants recalled their oral care experiences in two scenarios: for clients with dysphagia, and for clients without dysphagia [31]. Before the training, the participants reported the oral cleaning products they had used (including toothbrushes, toothpaste, mouthwash, and oral swabs) and the sites of oral cleaning (including the teeth, gums, tongue, and dentures) for their clients. Answers for these questions were “always”, “sometimes”, or “never”, which were awarded three, two, and one point, respectively. Therefore, the scores for these two questions ranged 4 to 12 points. In the third question, participants were asked how many times per day they provided oral cleaning for their clients.

#### 2.3.4. Validity and Reliability of Questionnaire

Content validity was verified by a panel of three experts (a nursing professor, a nursing supervisor, and a physician), who rated each questionnaire item on a scale of one to five; where five indicated the highest level of appropriateness and applicability. Items with a mean score of less than four were to be removed from the questionnaire. In this study, all original questionnaire items were retained with minor wording revisions. Reliability testing was conducted after the completed questionnaires were collected from the participants. The final questionnaire, and associated validity and reliability, are described in Table 1.

### 2.4. Ethical Considerations

Approval for data collection was obtained from the Research Ethics Committee of Cheng Ching Hospital’s Chung Kang Branch (HP 170036). All participants were given sufficient time to read the informed consent form and were provided with an opportunity to ask questions about the study.

### 2.5. Data Collection

A structured questionnaire was administered at the beginning and end of the one-day workshop. In order to receive a certificate of completion, all participants were required to email a self-report of oral hygiene skills (i.e., the oral cleaning products used, oral sites targeted for cleaning, and daily frequency of oral cleaning) three months later. To understand the subjective experiences of oral hygiene education, an interview guide was used via a telephone interview. The interview question required participants to provide their general feelings on the practicability of oral hygiene education to improve long-term care services during the three-month period between the delivery of the oral hygiene education program and the telephone interview. The entire telephone interview, which lasted an average of 8–10 min, was recorded and transcribed for further analysis. Data collection lasted from December 2017 to March 2018.

### 2.6. Data Analysis

The means and standard deviations of the total score regarding participants’ oral hygiene knowledge, attitudes, and skills, both pre- and post-delivery of the oral hygiene education program, were estimated for quantitative data analysis. The differences in means were then tested using paired t-tests. Cohen’s d was calculated as an indicator of the within-subject effect size [34]. The significance level was set at 0.05. Data were analyzed using the IBM SPSS Statistics Version 23.0 software (IBM, Armonk, NY, USA).

Qualitative data analysis included the transcription of the content of recorded interviews as original data; repeated readings of the transcripts to elicit meanings; and summary of the participants’ perspectives and feelings.

## 3. Results

### 3.1. Characteristics of the Study Population

A total of 80 long-term care workers (66 females and 14 males) participated in the oral hygiene education program. The older adults under the care of these participants included healthy, sub-healthy, frail, functionally dependent, and cognitively impaired individuals. Most of the participants (82.5%) had never received oral hygiene training, and over half of the participants were engaged in community-based services (51.2%), as shown in Table 2.

### 3.2. Effects of Oral Hygiene Education

#### 3.2.1. Oral Hygiene Knowledge and Attitudes

At the pre-program assessment of oral hygiene knowledge, none of the participants correctly answered that poor oral hygiene causes dry mouth (xerostomia) and bad breath (halitosis), in Items 7 and 10 of the structured questionnaire (Table 1), respectively. In addition, just one participant correctly answered that tooth loss was not a natural phenomenon of aging (Item 4). Only 12 participants understood the relationship between poor oral hygiene and aspiration pneumonia.

The proportion of participants who correctly answered the ten questions regarding oral hygiene knowledge increased in the post-program assessment. The mean score for the oral hygiene knowledge section in the pre-program assessment was 6.58 ± 1.23, and that in the post-program assessment was 7.32 ± 0.70. The mean scores for oral hygiene attitudes section were 2.37 ± 0.94 and 2.75 ± 0.57 in the pre- and post-program assessment, respectively.

The participants showed significant changes in their oral hygiene knowledge (*p* < 0.001) and attitudes (*p* = 0.001) after receiving oral hygiene education, as indicated by the paired t-tests in Table 3. The effect sizes for oral hygiene knowledge and attitudes were 0.60 and 0.41, respectively.

#### 3.2.2. Oral Hygiene Skills

Oral hygiene skills were evaluated based on the oral cleaning products used; the sites receiving oral cleaning; and the frequency of performing daily oral cleaning for clients before, and three months after, receiving oral hygiene education. In order to investigate whether the participants put their training into practice for the different client groups after returning to their workplaces, the results for clients with and without dysphagia are reported separately. Paired *t*-tests (Table 4) showed significant differences in the participants’ pre- and post-program scores of the daily frequency of oral hygiene skills use for clients with dysphagia; including the use of oral cleaning products (*p* = 0.005), oral cleaning sites (*p* < 0.001), and oral cleaning frequency (*p* < 0.001). The effect sizes for oral cleaning products, sites, and frequency were 0.33, 0.44, and 0.61, respectively.

For clients without dysphagia, paired t-tests (Table 4) showed significant differences in the participants’ pre- and post-program scores of daily oral cleaning frequency (*p* < 0.001) and the sites receiving oral cleaning (*p* = 0.044); with an effect size of 0.55 and 0.23, respectively. However, there was no significant difference between pre- and post-program scores in the frequency of use of oral cleaning products.

### 3.3. Experience and Suggestions of the Participants in Oral Hygiene Education

Six participants agreed to a telephone interview. The response rate was 75% (6/8). The participants described their experiences and suggestions regarding “clarifying misconceptions of oral health”, “improving staff experience”, and “identifying the importance of oral health”.

#### 3.3.1. Clarifying Misconceptions of Oral Health

The participants believed that mouth-rinsing may increase the risk of aspiration pneumonia in clients with dysphagia, whereas aspiration pneumonia was in fact induced by food residue left from meals. Participant 5 said, “I have increased the frequency and duration of mouth care for clients with dysphagia after receiving the oral hygiene training, because I know how to implement mouth care procedures carefully.” Reinforcing the frequency and duration of oral care for clients may increase staff workload; however, increased proficiency could facilitate comprehensive and timely performance of oral care procedures, thus decreasing future oral problems. Participant 2 said, “I became skillful through repetitive practice of oral care.” Long-term care workers should pay more attention to their clients’ oral hygiene because oral health has a significant impact on well-being and quality of life. Participant 3 said, “Since I know that pathogens in the oral cavity may induce systemic diseases, I implement oral hygiene very carefully for my clients every day.”

#### 3.3.2. Improving Staff Experience

Oral care is a reciprocal procedure between staff and clients in LTCSIs. Participant 1 said, “I perform oral hygiene procedures deliberately every day. The shining faces of my clients are a great encouragement for me. The clients are happy and appreciate my care, which leaves me feeling comfortable and vigorous. These experiences have created new meaning for me.” Participant 4 said, “I adjusted the oral hygiene procedure according to the clients’ conditions. This made me feel innovative and self-confident.”

#### 3.3.3. Identifying the Importance of Oral Health

In general, the participants felt ill-prepared to cope with the oral hygiene needs of clients with dysphagia, or neglected the oral hygiene of their clients. Therefore, the development of formal oral hygiene education programs for staff in long-term care services was essential. Participant 6 said, “I had no formal training on the oral hygiene of clients. I lacked oral hygiene knowledge for clients with or without dysphagia.”

## 4. Discussion

### 4.1. Oral Hygiene Knowledge

The findings of the 22-item questionnaire survey conducted before delivery of the oral hygiene education program suggest that long-term care workers may have significant gaps in their oral health knowledge. The findings of a previous 20-item questionnaire survey of oral health knowledge and practice among 53 registered nurses, conducted by Durgude et al., yielded similar results [31]. In contrast, a 19-item questionnaire survey of 106 nursing assistants’ oral-health knowledge, beliefs, and practice had previously shown that nursing assistants possess adequate oral hygiene knowledge for frail and functionally dependent older adults [30].

Aspiration pneumonia is the leading cause of hospitalization and death among clients receiving long-term care services [35]. Dysphagia is a recognized risk factor for aspiration pneumonia, but only causes pneumonia when combined with other factors, such as poor oral hygiene, periodontal disease and nocturnal denture wearing. Good oral hygiene care can prevent the incidence of periodontal disease and nocturnal denture wearing; therefore, oral hygiene plays a critical role in preventing aspiration pneumonia [8].

After undergoing the oral hygiene educational program, the participants understood the relationship between poor oral hygiene and aspiration pneumonia. The fact that good oral hygiene can reduce the incidence of aspiration pneumonia, as shown by a previous empirical study [6], should be highlighted and reiterated throughout the oral hygiene education program to reinforce the perceptions and behaviors of the participants. Given that less than one-fifth of participants in the current study had recently received oral hygiene training, it is apparent that providing up-to-date oral hygiene education opportunities to long-term care staff is an emergent issue.

### 4.2. Oral Hygiene Attitude

Nursing assistants and nurses are the first-line staff performing oral hygiene. Adequate educational training and the provision of the appropriate oral care products, support, and encouragement from the institutions will foster positive attitudes among these personnel and enable them to provide oral hygiene care in a confident and firm manner [13]. The qualitative interview data collected in this study identified that the participants evaluated the oral hygiene education positively. Further, the participants expressed confidence in their level of oral hygiene knowledge and their ability to properly conduct oral hygiene procedures, which reinforced their practical oral hygiene skills.

Qualitative interviews revealed that the participants increased their knowledge through oral hygiene education, and thereby increased their attitudes towards oral hygiene and their practical oral hygiene skills. In addition, performing the oral hygiene procedures elicited positive responses from their clients, which further motivated the participants to improve the oral health of their clients, thus creating a positive feedback loop. This research finding is contrary to that of Jablonski et al. [30], who examined the knowledge, beliefs, and practices of nursing assistants providing oral hygiene and found that their practice did not correspond to their knowledge. The results of the qualitative interview presented in the current study are essential and complementary to quantitative research, and are therefore an important aspect of the current research.

The present study shows that the oral hygiene education program can achieve significant improvements in the level of oral hygiene knowledge and attitudes among long-term care workers, to an extent sufficient to enable them to modify their oral care practice. The results are consistent with the literature, which reports that knowledge and attitudes are prerequisites for oral hygiene practice [36,37,38].

### 4.3. Oral Hygiene Skills

The present study explored the effects of implementing an oral hygiene education program on oral hygiene skills for clients with, or without, dysphagia. Daily oral hygiene procedures to remove dental plaque are a key factor in maintaining good oral health, as individuals with a high dental plaque index often have a high incidence of periodontal disease and an increased possibility of tooth loss [8]. However, clients in LTCSIs are often no longer able to perform oral hygiene procedures for themselves, and they must depend on nurses, nursing assistants, or other caregivers for such activities [20]. Mechanical and chemical cleaning are effective methods for removing dental plaque. Therefore, oral hygiene skills covered in the educational training program included oral cleaning products, sites, and the daily frequency of oral cleaning. In Taiwan, the assessment of plaque index falls within the professional domain of dentists and oral hygienists. Therefore, such an approach is not feasible in practice. Nevertheless, the findings of this study supported that the participants improved their oral hygiene skills, suggesting that removal of dental plague in clients could be reasonably expected.

In general, long-term care staff feel that performing oral hygiene is risky, due to the belief that using toothpaste and mouthwash may induce aspiration, especially in clients with dysphagia [17]. However, their misconceptions were clarified after receiving educational training, and significant progress in the frequency of their use of oral cleaning products and oral cleaning sites was observed. The significant increase in the frequency of oral cleaning for clients with or without dysphagia was a particularly positive outcome, as oral cleaning frequency is a key factor in maintaining oral health. Although the general recommendation is that oral cleaning should be performed once in the morning and once in the evening; cleaning after each of the three daily meals is ideal [28]. As shown in Table 4, the staff who underwent the oral hygiene education program performed oral cleaning close to three times a day, suggesting the oral hygiene educational program could help overcome barriers to the provision of oral hygiene procedures.

### 4.4. Lasting Effects of the Oral Hygiene Education

The effects of oral hygiene education are reportedly short-lived and unsustainable for long-term [20] or continuous effects [13]. Through combined qualitative and quantitative analyses, the present study shows that the oral hygiene education program significantly increased the participants’ daily oral cleaning frequency, products used, and cleaning sites for their clients at three months post-training. Therefore, the teaching content and methods (narration, demonstration, and teach-back) employed in this education program are practical and effective in oral hygiene education.

Oral hygiene education, like any other type of health education, encounters the problem of knowledge retention [39]. A short, focused oral hygiene education program had a significant impact on physician assistants’ retention rate of oral hygiene knowledge [40]. The key elements of improving learning retention rates include drawing the learner’s attention, stimulating interest in the topic, arousing motivation for learning, and building ways for learners to critically think about information [41]. The teaching activities in the oral hygiene education program include hosting narration, interactive multimedia presentation, demonstration, skills test, and teach-back to increase participant’s willingness to learn and think. Three months after receiving the training course, participants were required to provide photos of their practical application and relevant information, which motivated them to continue to perform oral hygiene care to their clients, to apply what they have learned, and to convert their ideas into reality to increase learning retention rates.

Another simple way to increase learning retention is through repetition of education [42]. Continuing education is among the factors that promote the growth of professional personnel. Previous studies demonstrated that the repetition and regular reinforcement of oral hygiene education can significantly improve retention of knowledge regarding oral hygiene [43,44,45]. Repetition of different evaluation procedures and/or oral hygiene motivation sessions may be a viable approach to the sustainability of oral hygiene knowledge, attitude, and behavior among long-term care staff over time.

### 4.5. Limitations

First, the present study was not randomized, and a single control group was used. Second, the three-month follow-up time may not have been adequate to detect long-term changes. Third, the oral cleaning products used, oral cleaning sites, and oral cleaning frequency reported at the three-month follow-up may have been affected by self-reporting bias. Rather than solely relying on self-reported surveys; various methods, including observation, should be used to objectively evaluate the effectiveness of oral cleaning. Fourth, it may be worth acknowledging that learning retention rates after the education program have not been evaluated in this study and should be included in future related studies. Finally, dental plaque index evaluations should be included in future practical tests of oral hygiene.

## 5. Conclusions

This study implemented an oral hygiene education training program, which was comprehensively evaluated based on self-reported data collected after three months of practical application of the taught oral hygiene skills, as well as qualitative interviews with the participants. Results of this pilot study indicate that oral hygiene education may be an effective method of improving oral hygiene knowledge, attitudes, and skills in staff providing long-term care services. The training program should be further evaluated, and, if proven to be successful and cost-effective in a wider range of trials, may be integrated in continuous professional education programs for long-term care staff. In this way, care providers will fully understand the importance of oral hygiene in older adults, and ensure best practice when providing oral care.

## Figures and Tables

**Table 1:** The structured questionnaire assessing oral hygiene knowledge, attitudes, and skills.

Part	Items	Scales	Reliability and Validity
Oral hygiene knowledge	1. The mechanical action of brushing teeth is the most effective to remove plaque.	One point was awarded for each correct “False” answer given for Items 4, 7, and 9. One point was awarded for each correct “True” answer given for the remaining seven Items. Scores ranged from 0–10 points, with higher scores indicating a higher level of oral hygiene knowledge.	The oral hygiene knowledge assessment was a dichotomous True/False test, and had a Kuder–Richardson coefficient of 0.90. The content validity of this questionnaire section was 0.92.
	2. Using dental floss before brushing teeth can enhance the cleaning effect and prevent periodontal disease.		
	3. Dentures have to be removed at night.		
	4. Tooth loss is a natural phenomenon in the aging process.		
	5. Poor oral hygiene may cause periodontal disease.		
	6. Poor oral hygiene may cause cardiac complications.		
	7. Poor oral hygiene is not related to xerostomia.		
	8. Poor oral hygiene may cause aspiration pneumonia.		
	9. Poor oral hygiene is not related to malnutrition.		
	10. Poor oral hygiene may cause halitosis.		
Oral hygiene attitudes	1. I will develop an individualized oral hygiene care plan for my clients.	One point was awarded for agreement with the statement and zero points were awarded for disagreement. Scores ranged from 0–3 points, with higher scores indicating a more positive attitude toward oral hygiene.	Oral hygiene attitudes were assessed with dichotomous Agree/Disagree statements, and had a Kuder–Richardson coefficient of 0.88. The content validity of this questionnaire section was 0.88.
	2. I know how to perform oral hygiene procedures for my clients every day.		
	3. I will try to improve the oral health status of my clients.		
Oral hygiene skills	1. Oral cleaning	Oral hygiene skills for clients with and without dysphagia were assessed with a paper and pencil exercise, where participants were first asked how many times a day they provided oral cleaning for their clients (Item 1). The following answer choices and scores were given for Items 2 and 3: “Always” (3 points), “Sometimes” (2 points), and “Never” (1 point). Therefore, each score for Items 2 and 3 in this section ranged from 4–12 points.	The Cronbach’s alpha of internal consistency for this questionnaire section assessing oral hygiene skills was 0.90, and the content validity was 0.90.
	Daily frequency		
	2. Oral cleaning products used daily		
	Toothbrush		
	Toothpaste		
	Oral Swabs		
	Mouthwash		
	3. Oral sites cleaned daily		
	Teeth Gums		
	Tongue Dentures		

Source: 1. Reference [30] and [31]. 2. Compiled by the authors.

**Table 2:** Demographic information (N = 80).

Variables	No (%)	Mean ± SD
Job title		
Nurses	25 (31.2)	
Nursing assistants	27 (33.8)	
Others (administrators and social workers)	28 (35.0)	
Education level		
Under junior college	26 (32.4)	
Above university	54 (67.6)	
Marital status		
Married	43 (53.8)	
Unmarried	37 (46.3)	
Received oral hygiene education		
Yes	14 (17.5)	
No	66 (82.5)	
Age		42.2 ± 13.0
Years in working		5.1 ± 9.3
Years’ experience in long-term care		2.6 ± 5.5
Long-term care service institution		
Home services	23 (28.8)	
Community-based services	41 (51.2)	
Institutional services	16 (20.0)	

Note: SD = standard deviation.

**Table 3:** Changes in oral hygiene knowledge and attitudes (N = 80).

Variable	Range	Pre-Program	Post-Program	Paired *t*	d *	*p*
		Mean ± SD	Mean ± SD			
Knowledge	0–10	6.58 ± 1.23	7.32 ± 0.70	5.24	0.60	<0.001
Attitude	0–3	2.37 ± 0.94	2.75 ± 0.57	3.55	0.41	0.001

Note: SD = standard deviation; d * = effect size calculated with Cohen’s d.

**Table 4:** Change in oral hygiene skills (N = 80).

Items	Pre-Program	Post-Program	Paired	d *	*p*
	Mean ± SD	Mean ± SD	*t* Value		
Clients with dysphagia					
Oral cleaning products	6.95 ± 4.06	8.32 ± 2.94	2.90	0.33	0.005
Toothbrush	1.64 ± 1.12	2.05 ± 0.98			
Toothpaste	1.70 ± 1.20	2.11 ± 0.93			
Oral swab	1.89 ± 1.14	2.18 ± 0.95			
Mouthwash	1.76 ± 1.19	1.96 ± 0.95			
Oral cleaning sites	7.61 ± 4.54	9.71 ± 3.31	3.96	0.44	<0.001
Teeth	1.86 ± 1.20	2.40 ± 0.88			
Gums	1.85 ± 1.24	2.37 ± 0.92			
Tongue	1.91 ± 1.21	2.40 ± 0.91			
Denture	1.99 ± 1.28	2.54 ± 0.89			
Oral cleaning frequency	1.96 ± 1.50	2.95 ± 1.50	5.41	0.61	<0.001
Clients without dysphagia					
Oral cleaning products	8.94 ± 3.34	9.20 ± 2.54	0.71	0.08	0.479
Toothbrush	2.50 ± 1.02	2.75 ± 0.68			
Toothpaste	2.54 ± 0.91	2.68 ± 0.76			
Oral swab	1.76 ± 1.08	1.51 ± 0.93			
Mouthwash	2.14 ± 1.02	2.26 ± 0.88			
Oral cleaning sites	9.51 ± 3.46	10.40 ± 2.87	2.05	0.23	0.044
Teeth	2.60 ± 0.89	2.74 ± 0.69			
Gums	2.34 ± 0.95	2.56 ± 0.87			
Tongue	2.36 ± 0.98	2.60 ± 0.82			
Denture	2.21 ± 1.10	2.50 ± 0.89			
Oral cleaning frequency	2.05 ± 1.36	2.93 ± 1.51	4.93	0.55	<0.001

Note: SD = standard deviation; d * = effect size calculated with Cohen’s d.
